# Development and validation of Egyptian developmental screening chart for children from birth up to 30 months

**DOI:** 10.7717/peerj.10301

**Published:** 2020-11-11

**Authors:** Ali M. El Shafie, Zein A.L. Omar, Mai M. Bashir, Sorour F. Mahmoud, Elsayedamr M. Basma, Ahmed E. Hussein, Alaa Mosad Mostafa, Wael A. Bahbah

**Affiliations:** 1 Department of Pediatrics, Menoufia Faculty of Medicine, Menoufia University, Shebin el-Kom, Menoufia, Egypt; 2 Ministry of Health Hospitals, Cairo, Egypt; 3 Department of Bioinformatics and Medical Statistics, Medical Research Institute, Alexandria University, Alexandria, Egypt

**Keywords:** Developmental delay, Egyptian Developmental Screening Chart, Screening tools, Validation, Z-score

## Abstract

**Background:**

Detecting developmental delay in children is an ongoing world commitment, especially for those below three years. To accurately assess the development of children; a culturally appropriate screening tool must be used. Egypt lacks such tool and multiple studies have shown that western tools are not suitable in other cultures.

**Objectives:**

To develop and validate an easy, rapid, culturally appropriate and applicable screening chart for early detection of developmental delay among Egyptian children from birth up to 30 months and develop a Z-score chart for motor and mental development follow up based on our Egyptian screening chart.

**Methods:**

A cross sectional randomized study was carried out on 1503 Egyptian children of both genders aged from birth up to 30 months assumed to have normal development according to the inclusion and exclusion criteria. They were selected from vaccination centers and well-baby clinics. Developmental milestones from Baroda development screening test (BDST) were applied on them after items were translated and adapted to Egyptian culture. Egyptian children developmental milestones scores were analyzed and carefully prepared in tables and charts. A 97% pass level of developmental achievements represents the threshold below which children are considered delayed. A Z-score chart for motor and mental development follow up was designed by calculating each age group achievement. The developed Egyptian developmental screening chart (EDSC) was validated against Ages and Stages Questionnaires (ASQ-3) as a reference standard in another different sample of 337 children in different age groups.

**Results:**

The developed EDSC is represented in a chart format with two curves 50% and 97% pass level. Children considered delayed when the score below 97% pass level. Results revealed a statistically significant difference between EDSC and BDST at 50% and 97% pass levels. A Z-score chart for motor and mental development follow up was designed by calculating each age group achievement. EDSC sensitivity and specificity were calculated 84.38 (95% CI [67.21%–94.72%]) and 98.36 (95% CI [96.22%–99.47%]) respectively with an overall test accuracy 97.03 (95% CI [94.61%–98.57%]) (*p* ≤ .001). Agreement between EDSC and ASQ-3 was high (kappa score was 0.827) with negative and positive agreement 98.36 and 84.38, respectively.

**Conclusions:**

Extensive revision of the BDST was needed in order to create and validate a more culturally appropriate Egyptian screening chart. This is the first study to create and validate an Egyptian-specific screening tool, to be rapid and easy to use in Egypt for early detection of developmental delay and enabling early intervention practices. A Z-score curve is reliable for follow up motor and mental development by calculating each age group achievement.

## Introduction

Almost 200 million children worldwide suffer from different forms of disability, the majority of them present in developing countries. Children in low and middle-income countries are at risk for not fulfilling their potential for physical and mental development due to poverty and other risk factors as malnutrition (Fischer, Morris & Martines, 2014; Black et al., 2017).

Developmental assessment of young children is a challenging task. Relying exclusively on clinical judgment alone may be misleading (Council on Children With Disabilities et al., 2006). Thus, screening tools are important to identify children for further testing and follow-up. A screening tool may be feasibly administered to the parents or tested on the child. Parent-administered screening tools are of great value especially in cases of children’s sleepiness, irritability and illness. The range of sensitivity and specificity of 70% to 80% has been considered suitable for developmental screening tools (Urkin, Bar-David & Porter, 2015; Oberklaid & Drever, 2011).

Developmental screening is indicated whenever a problem is noticed during developmental surveillance or when doubts are raised by parents, caregivers or child health practitioners. It is more accurate when applying standardized assessments of children’s developmental status rather than simple clinical impressions. The American academy of pediatrics recommended administration of standardized screening tools at the ages (9, 11, 24, or 30 months) in order to produce effective developmental surveillance. It also recommends that performing repeated developmental screening is more accurate and reliable than single assessment (Bright Futures Steering Committee & Medical Home Initiatives for Children With Special Needs Project Advisory Committee, 2006; Lipkin & Macias, 2020).

One of the effective screening tools is the Baroda development screening test (BDST) by Phatak and Khurana (Phatak & Khurana, 1991). It is a simple, rapid, and cost-effective tool. BDST checklist contains 54 items selected from the norms giving in Bayley Developmental Screening Test of infants (Baroda norms) (Bayley, 1969; Bell & Allen, 2000). Baroda screening test considered valid in field survey, as well as clinical practices with sensitivity and specificity 95%–65% in sequence (Phatak & Khurana, 1991).

There are other screening tools created in high income countries such as the Ages and Stages questionnaire (ASQ) which is a parent report tool designed and developed by Squires, Bricker & Twombly (2009). The ASQ consisting of 21 questionnaires (30-items each) spanning the age of 2–60 months, with an overall sensitivity of 75% and specificity of 86% (Singh, Yeh & Blanchard, 2017). Another one, Denver Developmental Materials II (formerly DDST), was developed to be used by professionals or trained paraprofessionals to determine if a child’s development is within the normal range (Drachler et al., 2005). Other tools developed in low- and middle-income countries (LMIC) include the Trivandrum Developmental Screening Chart (TDSC), which is simple, short and requires limited training for identifying children who have developmental delays up to 2.5 years (Fischer, Morris & Martines, 2014; Nair et al., 1991). The Guide for Monitoring Child Development in Turkey is another screening tool described as a brief, open-ended, pre-coded interview with the primary caregiver for children from 0 to 2 year(s) of age (Ertem et al., 2008), as well as the Malawian Developmental Assessment Tool which revealed a good validity in targeting children from birth up to six years (Gladstone et al., 2008).

This study aims to develop and validate an easy, rapid, culturally appropriate and applicable screening chart for early detection of developmental delay among Egyptian children from birth up to 30 months and develop a Z-score chart for children follow up based on our Egyptian screening chart.

## Materials & Methods

The study was conducted in two steps to develop and validate the Egyptian developmental screening chart (EDSC) from January 2019 till January 2020 in Egypt. The University of the Menoufia granted Ethical approval to carry out the study within its facilities (Ethical Application Ref: jm420-c5a3d, Institutional Review Boards IRB Approval ID: 180112Ped). Written consent was obtained from parents/or guardians who were informed about the objective of the study, its benefits and the absence of any risk associated with the participation of their children.

### Step 1: Instrument development

#### Participants

A cross sectional randomized study was implemented on 1,503 normally developed Egyptian children aged from birth up to 30 months at vaccination centers and well-baby clinics. The minimum sample size of 1,500 children was calculated as adequate sample required to perform the study assuming a significance level of 95% (α = 0.05), and statistical power (1–β) of 80% (Daniel, 1991; Killeen, 2005). The sample size was calculated according to Charan & Biswas (2013). Online Open Source Epidemiologic Statistics for Public Health was also used to confirm the calculation (Dean, Sullivan & Soe, 2013).

Full term children from birth up to 30 months of age with anthropometric measurements (weight, length/ height and head circumference) within normal range for age according to WHO growth charts were included in the study (WHO Multicentre Growth Reference Study Group, 2006). Any child had history of prematurity, hospital admission including neonatal intensive-care unit (NICU), low socioeconomic level, malnourished according to WHO (≤-2 standard deviation of weight to length/ height), chronic diseases (cardiac, hematological, chest or endocrine diseases) and developmental or physical disabilities was excluded.

A total number of 1,600 children were enrolled in the study. Exclusion criteria were applied to 97 children leaving 1503 children as a final total sample to be included in the study. The selected Children were divided into 30 groups based on their chronological age (CA), with each age group consisted of 44 to 58 children.

#### Milestones and chart development

EDSC checklist based on BDST questionnaire. BDST questionnaire consists of 54 items 22 motor (gross and fine motor) and 32 mental (cognitive, social and language). These items were chosen carefully from Bayley scale of infants which consist of 230 items (67 motor and 163 for mental development). Milestones were arranged from 0 to 30 months of age in an ascending order. Our research team discussed the cultural appropriateness of Baroda items and translated it to Arabic. Then items were clarified to the parents/or guardians with their local expressions till parents/or guardians could easily understand and answer unequivocally. A training workshop was provided for the field staff to explain the items on the checklist and how to conduct an interview with the parents/or guardians. A pilot study of 150 children was designed (five children per month) to test all items of the developmental checklist for Egyptian children and also to test and standardize the capabilities of the involved team before proceeding to the main data collection. The pilot study concluded that the data collector team was able to understand and apply the items, parents were apple to understand and answer questions easily with yes or no, items didn’t return with missing answers and there was a certain degree of variability in most of items.

The scores of checklist items passed by children were analyzed and tabulated. 97% pass level of developmental scores of children was taken as a reference. The 50% and 97% level age placement of each item were plotted against its corresponding CA of children and then smoothed into two curves. Any child score below 97% pass level considered delayed.

A Z-score chart for motor and mental development follow up was designed also by calculating each age group achievement.

#### Measurements and data collection

Socioeconomic and demographic factors were collected using Fahmy schedule which is used for estimating socioeconomic standard in Egypt (Fahmy et al., 2015). Low socioeconomic status was excluded as it has a negative environmental influence on child development, for example; malnutrition can influence development by causing him or her to fuss more or play less and affect brain development function (Prado & Kathryn, 2014), also Poverty and social-cultural factors increase both physiological and behavioral deficits (Fernald et al., 2017).

Children were examined for any developmental or physical disability. Weight, recumbent length (for less than 24 months), height (from 24 months to 30 months) and head circumference (HC) were measured. Weight was measured by (LAICA model bf 2051, Italy) till the age of 2 years then another scale (Beurer model GS 11, Germany) was used till age of 30 month. The length of children was measured by a recumbent baby length scale. The height was measured by Harpenden fixed stadiometer. HC was measured by flat metal tape. This is followed by an interview to their parents/or guardians to complete the developmental checklist.

### Step 2: Validation of EDSC

A validation study to EDSC was done against ASQ-3 as a gold standard tool (Squires, Bricker & Twombly, 2009). A different sample of 337children were enrolled in a cross sectional randomized study from vaccination centers and well-baby clinics. A sample size of 299 children was calculated as enough required sample to conduct this agreement study, assuming that all individual but one pair agree with each other (Liao, 2010). A total number of 345 children were enrolled in the study. Exclusion criteria were applied to 8 children leaving 337 children as a final total sample. The selected children were divided into 15 groups based on their CA (first 15 age groups in the ASQ-3) ranging from 2 to 30 months. Children suffered from acute severe illness or previously diagnosed with a developmental disorder were excluded.

EDSC checklist was applied to the parents/or guardians and the score was calculated by one of our team work. After obtaining informed consent, a detailed clinical evaluation was done. 97% pass level is determined as a cut off point, any child failed to pass above the 97% criterion was defined as ‘delayed’. ASQ-3 was also applied to the same participants as a reference standard by another observer in our team who was blinded to the results of EDSC. ASQ-3 questionnaire contains 30 questions for each specific age group. These questions examine five domains: Fine Motor, Gross Motor, Communication, Problem-Solving and Personal-Social; each domain includes 6 questions that can be answered with a yes (10 points), sometimes (5 points) or not yet (0 points), as well as nine open-ended questions. Scores falling in the white area indicate the child is developing typically. Scores falling in the gray area mean the child should be monitored and another screening may be needed later on. Scores falling in the black area (cut off point) mean that the child may be at risk for developmental delay and should be referred for further assessment (2 SD below mean).

### Statistical analysis

Data were collected and entered into the computer using SPSS (Statistical Package for Social Science) a program for statistical analysis (version 21) (IBM Corp., Armonk, NY, USA). Data were entered as numerical or categorical, as appropriate and described using minimum, maximum, mean, standard deviation. Categorical variables were described using frequency and percentage. Comparisons were carried out between two studied dependent (developmental age of the Egyptian children on 50% and 97% pass level using Baroda curve vs the developmental age of the same child using EDSC) normally distributed variables using paired *t*-test (Box, 1987). A Z-score was calculated for each age group at the following: - 3, -2, -1, 0,1,2,3 equally in sequence the percentiles (0.2nd, 2.3rd, 16th, 50th, 84th, 97.7th, 99.8th respectively) (Wang & Chen, 2012). Polynomial trend line curves were used by Microsoft Excel (Microsoft Office Professional). (Hargreaves & McWilliams, 2010)

Validation evaluation was carried out using MedCalc Software version 14 (DeLong, DeLong & Clarke-Pearson, 1988). The following tests were carried out: Sensitivity (true positive rate), Specificity, positive and negative predictive value as well as accuracy (Zhou, McClish & Obuchowski, 2009). Kappa values interpretations 0.75 considered as excellent, 0.40 to 0.75 as fair to good, and below 0.40 as poor according to fleiss’s equally arbitrary guidelines (Fleiss, 1981). An alpha level was set to 5% with a significance level of 95%.

## Results

**Figure 1:**
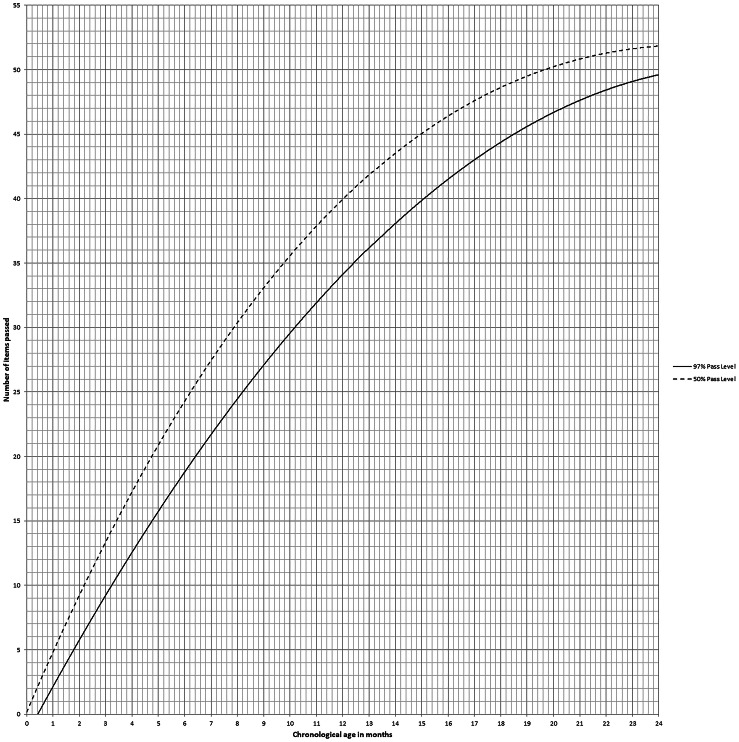
Shows 50% and 97% pass level where chronological age plotted horizontally and number of items passed plotted vertically.

A total sample of 1503 children were enrolled in EDSC design, 785 (52.2%) of them were males and 718 (47.8%) were females. The socioeconomic standard of the participants was high in 1076 (71.6%) and moderate in 427 (28.4%) children. The Egyptian screening chart’s vertical line indicates the number of items passed plotted against the CA on the horizontal one. The 50% pass level curve drawn intermittently, whereas the 97% pass level curve drawn continuously. Any child score below the continuous line was considered developmentally delayed. Developmental age (DA) can be calculated from EDSC by intersection of the horizontal level of the score with the 50% pass level curve. Also, Developmental Quotient (DQ) can be calculated directly from the EDSC by the equation (DQ = (DA/CA)× 100) (Fig. 1).

**Figure 2:**
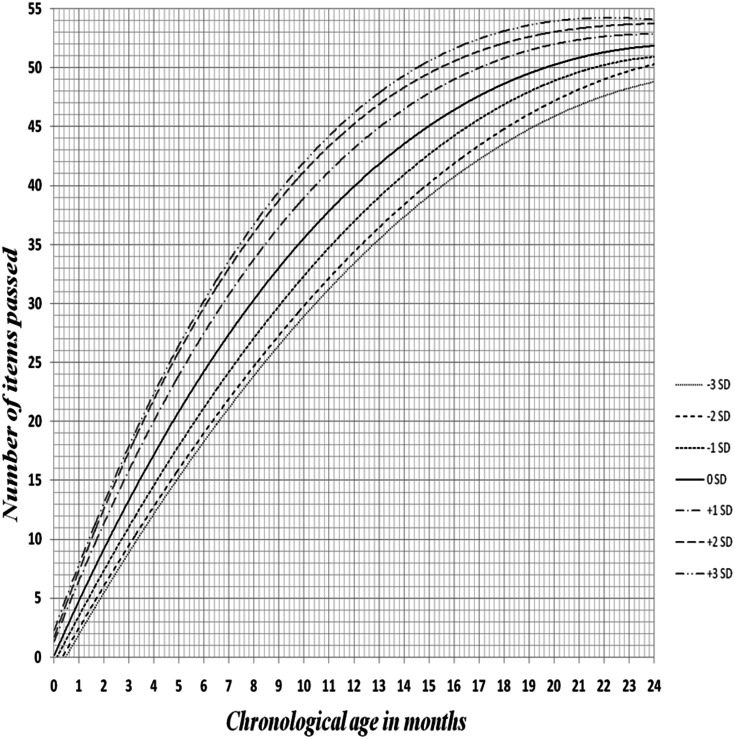
Z-score curve of Egyptian developmental screening chart of infants showing the age placement of each item at various percentage pass levels up to 24 months.

Statistically, there was a significant difference between EDSC of children compared with BDST, with *p*-value ≤0.001, calculated by measuring the Egyptian children developmental age on both charts at 50% and 97% passing levels. (Table 1). A Z-score curve of EDSC for children demonstrated relevant age placement of each item at various percentage passing levels and any child performance plotted above -2SD curve was considered normal, while who recognized below -2SD was deemed developmentally delayed .The Z-score chart also can be used for motor and mental development follow up (Fig. 2).

Validation of EDSC against ASQ-3 (Reference standard) was assessed in a different sample of 337 children 173 (51.3%) females and 164 (48.7%) males. Child’s score lie below 97% pass level was considered “EDSC delay” (Tool positive). The sensitivity and specificity of EDSC was found 84.38 (95% CI [67.21%–94.72%]) and 98.36 (95% CI [96.22%–99.47%]) respectively with an overall test accuracy 97.03 (95% CI [94.61%–98.57%]) (*p* ≤ .001). Negative and positive agreement between EDSC and ASQ were 98.36 and 84.38 respectively (Table 2). When suspected cases were considered as delayed, the calculated kappa measure of agreement between EDSC and ASQ-3 was 0.827 (95% CI [0.723–0.932]) (*p* = 0.000) (Table 3).

**Table 1:** Comparison between developmental age of Egyptian and Baroda charts at 50% and 97% pass level.

**Age category in months**	**DA 50%**		***P* value**	**DA 97%**		***P* value**
	**BARODA**	**EGYPTIAN**		**BARODA**	**EGYPTIAN**	
1.0	0.48 ± 0.405	0.86 ± 0.284	*t* = 8.874 *p* = 0.000*	1.80 ± 0.319	1.53 ± 0.339	*t* = 22.844 *p* = 0.000*
2.0	2.23 ± 0.697	2.73 ± 0.751	*t* = 35.133 *p* = 0.000*	4.12 ± 0.955	3.71 ± 0.848	*t* = 18.920 *p* = 0.000*
3.0	2.45 ± 0.540	3.00 ± 0.571	*t* = 47.364 *p* = 0.000*	4.48 ± 0.744	4.00 ± 0.631	*t* = 22.862 *p* = 0.000*
4.0	3.87 ± 0.896	4.48 ± 0.895	*t* = 55.874 *p* = 0.000*	6.18 ± 0.999	5.67 ± 1.027	*t* = 24.461 *p* = 0.000*
5.0	4.26 ± 0.779	4.89 ± 0.768	*t* = 73.817 *p* = 0.000*	6.64 ± 0.795	6.15 ± 0.873	*t* = 22.117 *p* = 0.000*
6.0	6.25 ± 0.812	7.00 ± 1.018	*t* = 14.777 *p* = 0.000*	8.89 ± 0.986	8.55 ± 1.055	*t* = 15.113 *p* = 0.000*
7.0	6.30 ± 0.974	7.13 ± 1.222	*t* = 13.742 *p* = 0.000*	8.95 ± 1.206	8.64 ± 1.287	*t* = 13.864 *p* = 0.000*
8.0	7.29 ± 0.844	8.40 ± 1.012	*t* = 15.891 *p* = 0.000*	10.17 ± 1.015	9.97 ± 1.124	*t* = 7.724 *p* = 0.000*
9.0	8.10 ± 1.077	9.16 ± 1.198	*t* = 24.003 *p* = 0.000*	11.11 ± 1.250	11.00 ± 1.390	*t* = 3.880 *p* = 0.000*
10.0	9.55 ± 0.835	10.81 ± 1.016	*t* = 46.485 *p* = 0.000*	12.85 ± 1.045	12.92 ± 1.143	*t* = 3.012 *p* = 0.000*
11.0	10.66 ± 1.077	12.16 ± 1.288	*t* = 47.653 *p* = 0.000*	14.27 ± 1.350	14.47 ± 1.518	*t* = 5.337 *p* = 0.000*
12.0	11.30 ± 1.144	12.92 ± 1.332	*t* = 57.061 *p* = 0.000*	15.06 ± 1.379	15.35 ± 1.581	*t* = 7.141 *p* = 0.000*
13.0	11.49 ± 1.408	12.54 ± 1.625	*t* = 14.308 *p* = 0.000*	15.06 ± 1.705	15.42 ± 1.809	*t* = 10.663 *p* = 0.000*
14.0	12.34 ± 1.403	13.24 ± 1.637	*t* = 10.463 *p* = 0.000*	15.84 ± 1.809	16.33 ± 1.862	*t* = 12.790 *p* = 0.000*
15.0	13.13 ± 1.396	14.28 ± 1.733	*t* = 15.038 *p* = 0.000*	17.03 ± 2.161	17.49 ± 1.975	*t* = 10.424 *p* = 0.000*
16.0	14.13 ± 1.555	15.15 ± 1.924	*t* = 14.768 *p* = 0.000*	18.42 ± 2.580	18.78 ± 2.096	*t* = 4.107 *p* = 0.000*
17.0	15.31 ± 1.656	16.64 ± 1.771	*t* = 26.917 *p* = 0.000*	20.28 ± 2.871	20.40 ± 2.002	*t* = 0.873 **p* = 0.387 NS*
18.0	15.88 ± 1.606	17.44 ± 1.893	*t* = 22.052 *p* = 0.000*	21.18 ± 2.525	20.99 ± 1.726	*t* = 1.259 **p* = 0.215*
19.0	17.07 ± 1.544	18.24 ± 1.712	*t* = 15.385 *p* = 0.000*	22.93 ± 2.267	22.51 ± 1.827	*t* = 2.888 *p* = 0.006*
20.0	17.89 ± 1.946	19.20 ± 2.423	*t* = 11.169 *p* = 0.000*	23.78 ± 2.770	23.14 ± 2.249	*t* = 3.621 *p* = 0.001*
21.0	17.94 ± 1.705	19.20 ± 2.155	*t* = 11.397 *p* = 0.000*	24.04 ± 2.102	23.59 ± 2.092	*t* = 3.359 *p* = 0.001*
22.0	18.08 ± 1.687	19.40 ± 2.188	*t* = 11.174 *p* = 0.000*	24.25 ± 2.262	23.55 ± 2.031	*t* = 4.320 *p* = 0.000*
23.0	18.43 ± 1.932	19.92 ± 2.700	*t* = 9.816 *p* = 0.000*	24.08 ± 2.358	23.74 ± 2.326	*t* = 3.461 *p* = 0.001*
24.0	19.19 ± 1.671	21.02 ± 2.692	*t* = 10.816 *p* = 0.000*	25.18 ± 2.484	24.61 ± 2.198	*t* = 4.316 *p* = 0.000*
25.0	19.40 ± 1.525	21.02 ± 2.379	*t* = 9.360 *p* = 0.000*	25.31 ± 1.969	25.25 ± 2.104	*t* = 1.250 **p* = 0.218 NS*
26.0	19.42 ± 1.496	21.13 ± 2.477	*t* = 9.644 *p* = 0.000*	25.33 ± 1.674	25.33 ± 2.168	*t* = 1.226 **p* = 0.226 NS*
27.0	20.20 ± 1.145	22.43 ± 2.296	*t* = 11.880 *p* = 0.000*	25.78 ± 1.954	26.37 ± 1.764	*t* = 0.709 **p* = 0.482 NS*
28.0	20.19 ± 1.331	22.38 ± 2.407	*t* = 10.940 *p* = 0.000*	25.96 ± 1.846	26.53 ± 1.930	*t* = 3.516 *p* = 0.001*
29.0	20.45 ± 1.306	23.10 ± 2.337	*t* = 13.136 *p* = 0.000*	26.76 ± 1.732	27.12 ± 1.914	*t* = 3.649 *p* = 0.001*
30.0	21.03 ± 0.969	24.37 ± 1.549	*t* = 30.542 *p* = 0.000*	24.94 ± 3.203	27.35 ± 1.857	*t* = 0.837 **p* = 0.411 NS*

## Discussion

Child assessment in developed countries often uses Western developmental tools (e.g., Bayley scales and the Denver II), which have been designed and validated in Western countries and have been used in non-Western or low and middle-income (LAMI) countries only by translation to another languages (Ertem et al., 2008; Gladstone et al., 2008). These translations may not meet local typicality and culture specificity leading to misinterpretation of the results (Sabanathan, Wills & Gladstone, 2015). For example, all domains of Western tests have some items that are culturally inappropriate for rural Africa, such as prepare ‘cereal’, ‘play board games’ and other uncommon activities (Gladstone et al., 2008).

Lack of appropriate instruments in low and middle income countries is a major barrier to monitor child development (Engle et al., 2007). LMIC tried to adopt internationally standardized tests that have been proven to measure a construct of child development through time and across cultural diversity (Amod, Cockcroft & Soellaart, 2007).

**Chunsuwan and Hansakunachai** (Chunsuwan, Hansakunachai & Pornsamrit, 2016) claimed that using instruments developed mainly from a single culture may not provide the same results with another due to the cultural influence, which is called a deviant development (Duc, 2016; Toh et al., 2017). Also, other studies have contended the importance of making more efforts in the development of screening tools that respect the local differences (Fernald et al., 2017).

A developmental screening tool for community should be simple, cost-efficient, less time consuming, valid and easy to understand by health workers and parents. The tool should consider cultural differences and reflect all developmental domains (Nair et al., 2013; Chopra, Verma & Seetharaman, 1999).

BDST have been used in Egypt for children motor and mental development assessment as a rapid, easy and valid test according to many studies (Fischer, Morris & Martines, 2014; Robertson et al., 2012). Items of the Indian BDST found to be simple, applicable and convenient to Egyptian society. If the child performance lies below the 97% pass level, it represents the vulnerable population that requires further investigation for developmental delay (3%). There was a significant difference between EDSC and BDST, this was identified by measuring the DA of children in either chart 50% and 97% passing level. So establishing an Egyptian developmental chart based on Egyptian norms would be more suitable to the Egyptian cultural context.

**Table 2:** Test characteristics of EDSC against ASQ, having “EDSC Delay” as tool positive.

Criteria of test positive	Child delayed in EDSC taken “EDSC delay” **(Tool positive)**
Sensitivity^a^	84.38 (95% CI [67.21%–94.72%])
Specificity^b^	98.36 (95% CI [96.22%–99.47%])
Positive Predictive Value^c^	84.38 (95% CI [69.09%–92.88%])
Negative Predictive value^d^	98.36 (95% CI [96.41%–99.26%])
Overall Test Accuracy^e^	97.03 (95% CI [94.61%–98.57%]) (*p* ≤ .001)
**Proportions of specific agreement:**	
Negative agreement	= 2 * 300 / (2*300 + 5 +5) = 98.36%
Positive agreement	= 2 * 27 / (2*27 + 5 +5) = 84.38%

**Notes.**

a *Sensitivity*: probability that a test result will be positive when the disease is present (true positive rate).

b *Specificity*: probability that a test result will be negative when the disease is not present (true negative rate).

c *Positive predictive value*: probability that the disease is present when the test is positive.

d *Negative predictive value*: probability that the disease is not present when the test is negative.

e *Accuracy*: overall probability that a patient is correctly classified.

**Table 3:** Agreement between Egyptian Developmental Screening Chart (EDSC) and ASQ-3 Questionnaire.

		**ASQ Grade** **(Standard)**		
		**Under-developed**	**Normal**	**Total**
**Grade on Egyptian Developmental Screening Test**	**Under-developed**	(TP)	(FP)	
		27	5	32
		(8.01%)	(1.48%)	(9.50%)
	**Normal**	(FN)	(TN)	
		5	300	305
		(1.48%)	(89.02%)	(90.50%)
	**Total**	32	305	337
		(9.50%)	(90.50%)	(100.0%)
**Kappa**		0.827		
**Standard error**		0.053		
*p* value		0.000^*^	
**Weighted kappa**		0.827		
**Standard error**		0.053		
**95% CI**		0.723 to 0.932		

**Notes.**

* Significant difference means *P*- value < 0.05.

TP, True positive; FP, False positive; FN, False negative; TN, True negative

The EDSC is a sensitive and reliable screening test for developmental delay in infants and young children. It is not time-consuming, special test equipment is not needed, and developmental milestones need not be strictly memorized by the parents. The chart design is simple and conceptually clear for physicians and parents to demonstrate the general development of a child, whether normal or delayed. At follow-up, it is useful in portraying a child’s continued progress or lack of progress.

A Z-score chart was developed to facilitate follow up of children motor and mental development. Any child’s score below -2SD considered developmentally delayed and need follow-up for child’s progress in future visits. Developmental screening tools of other nations didn’t mention any trial to develop a Z-score chart for developmental follow up. So this study is the first study that had established developmental screening tool in the form of Z-score chart for follow up.

Validation of EDSC took place on other participants (either normal or delayed) against ASQ-3 which was the most appropriate gold standard for these age groups as it’s a feasible screening tool, inexpensive, easy to use, and was appreciated by the parents (Elbers et al., 2008). What is more; Validity of ASQ-3 has been examined across different countries with an overall sensitivity of 75% and specificity of 86% (Singh, Yeh & Blanchard, 2017). The range of sensitivity and specificity of 70% to 80% have been considered suitable for developmental screening tools (Urkin, Bar-David & Porter, 2015; Oberklaid & Drever, 2011). ASQ-3 was found as a valid and reliable as a developmental screening tool in Egypt, this supported the idea of using it as a reference standard tool (EL-Ella et al., 2017).

In this study, the EDSC sensitivity was found to be 84.38% with specificity 98.36%. EDSC was developed as a screening tool for developmental delay; as test positive predictive value of 84.38%. In this position, one item delay as test positive gives an excellent ‘Negative Predictive Value’ of 98.36% which is acceptable for a screening tool. A perfect screening test should be with a high sensitivity, high negative predictive value and not having much compromise on specificity, EDSC fulfills these criteria.

## Conclusions

EDSC is valid, easy, rapid and culturally appropriate tool that facilitates early detection of developmental delay in children by pediatric practitioners and health workers. Subsequently, we may stress the idea that each country build up its own screening tool. Furthermore, developing a Z-score chart renders a rapid and reliable chart to use at the follow-up stages of the Egyptian children motor and mental development.

##  Supplemental Information

10.7717/peerj.10301/supp-1 Supplemental Information 1 Baroda developmental screening test checklist

10.7717/peerj.10301/supp-2 Supplemental Information 2 Arabic checklist

10.7717/peerj.10301/supp-3 Supplemental Information 3 Results

10.7717/peerj.10301/supp-4 Supplemental Information 4 Designing Raw Data

10.7717/peerj.10301/supp-5 Supplemental Information 5 Validation data

## Additional Information and Declarations

### Data Availability

The following information was supplied regarding data availability: Data are available as a Supplemental File.
