# Supplementary material for: Development and validation of Egyptian developmental screening chart for children from birth up to 30 months
Source: PeerJ. 2020 Nov 11;8:e10301. doi: 10.7717/peerj.10301 (PMC7666562; doi:10.7717/peerj.10301)
Supplement: Supplemental Information 1 [file peerj-08-10301-s001.docx]

| Age Items Response | | |
| --- | --- | --- |
| 1^st^ month | 1. ARMS & Legs thrust in play | Yes NO |
|  | 1. momentary regard | Yes NO |
|  | 1. lateral head movement (prone) | Yes NO |
| 2^nd^ month | 1. responds to sound | Yes NO |
|  | 1. Follows moving person | Yes NO |
|  | 1. Free inspection of surrounding | Yes NO |
| 3^rd^ month | 1. Social smile/vocalizes | Yes NO |
|  | 1. Eye co-ordination | Yes NO |
|  | 1. Head erect &steady | Yes NO |
| 4^th^ month | 1. Holds head steady | Yes NO |
|  | 1. Recognizes mother | Yes NO |
|  | 1. Elevates on arms | Yes NO |
| 5^th^ month | 1. Play with rattle / hand play | Yes NO |
|  | 1. Reaches for dangling ring | Yes NO |
|  | 1. Sits with slight support | Yes NO |
| 6^th^ month | 1. Turns head to sounds | Yes NO |
|  | 1. Turns from back to side | Yes NO |
|  | 1. Exploitive paper play | Yes NO |
| 7^th^ month | 1. Discriminates strangers | Yes NO |
|  | 1. Pulls to sit | Yes NO |
| 8^th^ month | 1. Bangs in play | Yes NO |
|  | 1. Sits alone steadily | Yes NO |
| 9^th^ month | 1. Retails two things in two hands | Yes NO |
|  | 1. Pulls to stand | Yes NO |
|  | 1. Playful response to mirror image | Yes NO |
|  | 1. Sits with good co-ordination | Yes NO |
| 10^th^ month | 1. Pulls string-secures toys | Yes NO |
|  | 1. Co-operates in play | Yes NO |
|  | 1. Crawling (pre walking) | Yes NO |
| 11^th^ month | 1. Rings bell purposefully | Yes NO |
|  | 1. Fine prehension | Yes NO |
|  | 1. Raises to sit | Yes NO |
|  | 1. Stands by furniture | Yes NO |
| 12^th^ month | 1. Adjust two words | Yes NO |
|  | 1. Says da-da | Yes NO |
| 13^th^ to 15^th^ month | 1. Inhibits on command | Yes NO |
|  | 1. Midline skills | Yes NO |
|  | 1. Walks with help | Yes NO |
|  | 1. Turns pages | Yes NO |
| 16^th^ to 18^th^ month | 1. Imitates words | Yes NO |
|  | 1. Stands alone | Yes NO |
|  | 1. Spontaneous scribble | Yes NO |
|  | 1. Throws balls | Yes NO |
|  | 1. Aufstein 1. | Yes NO |
|  | 1. Walks alone | Yes NO |
|  | 1. Gestures for wants | Yes NO |
| 19^th^ to 24^th^ month | 1. Shows shoes, etc. | Yes NO |
|  | 1. Two words | Yes NO |
|  | 1. Walks up &down stairs with help | Yes NO |
|  | 1. Words for wants | Yes NO |
| 25^th^ to 30^th^ month | 1. Two words sentences | Yes NO |
|  | 1. Names three objects | Yes NO |
|  | 1. Stands on one foot | Yes NO |
|  | 1. Walks up & down stairs without help | Yes NO |

**CHECK LIST OF EGYPTIAN DEVELOPMENTAL SCREENING CHART FOR INFANTS FROM BIRTH UP TO 30 MONTHS.**
