# Supplementary material for: Development and validation of Egyptian developmental screening chart for children from birth up to 30 months
Source: PeerJ. 2020 Nov 11;8:e10301. doi: 10.7717/peerj.10301 (PMC7666562; doi:10.7717/peerj.10301)
Supplement: Supplemental Information 2 [file peerj-08-10301-s002.docx]

|  | | **بيانات التطور العقلى والحركى :** | | | | | |
| --- | --- | --- | --- | --- | --- | --- | --- |
|  | |  | | | | | |
|  |  | | **العمر البند** | | | | |
| **نعم لا** | | | | **1-يحرك يداه وقدميه علي الجانبين (يشقل بايده ورجله)** | | **شهر** |  |
| **نعم لا** | | | | **2-يركز بعينيه علي وجه الام للحظات او (الضوء)** | |  |  |
| **نعم لا** | | | | **3-يحرك رأسه للجانبين وهو نائم علي بطنه** | |  |  |
|  | | | | | | | |
| **نعم لا** | | | | **4-يتخض او يسكت عند سماع الأصوات العاديه مثل(الكلام،الشخشيخه)** | | **شهران** |  |
| **نعم لا** | | | | **5-يتابع بعينيه الام عندما تتحرك او عند تحريك لعبه امامه** | |  |  |
| **نعم لا** | | | | **6-بيحرك عينيه ليكتشف المكان حوله.محاولا ملاحظة شئ أمامه.** | |  |  |
|  | | | | | | | |
| **نعم لا** | | | | **7-يبتسم وبيناغي بصوت واضح عند الكلام معه أو أصدار أصوات له.** | | **3 أشهر** |  |
| **نعم لا** | | | | **8-هل الطفل قادر علي النظر حوله في كل الاتجاهات (لاعلي- أسفل،يمينا-يسارا).** | |  |  |
| **نعم لا** | | | | **9-يرفع رأسه لفتره قصيره(بترنح) عندما تحمله الام علي كتفها** | |  |  |
|  | | | | | | | |
| **نعم لا** | | | | **10-يرفع رأسه بثبات عاليا عند جذبه من زراعيه ليجلس أو رفعه قائما من أسفل الباط .** | | **4 شهور** |  |
| **نعم لا** | | | | **11-يبتسم عند رؤيه امه، يهدأ عندما تأتي.** | |  |  |
| **نعم لا** | | | | **12-عندما ينام علي بطنه هل يكون قادر علي رفع صدره من علي السرير بالأستناد علي مرفقه أو يرفع جسمه باأستناد عي ذراعه.** | |  |  |
|  | | | | | | | |
| **نعم لا** | | | | **13-عندما توضع شخشيخه بيده هل الطفل ينظر إليها،يضعها بفمه،يهزها أو يلعب بها بأي طريقه أخري.** | | **5 شهور** |  |
| **نعم لا** | | | | **14-هل الطفل يبدي إهتمام بالأشياء حوله ويحاول الوصول لها بمد ذراعه(لايشترط أن يستطيع الوصول لها)** | |  |  |
| **نعم لا** | | | | **15-يجلس مستند علي وساده او ذراع الام عندما تكون القدم مفروده. يمكن أن تختبر بواسطه الطبيب.** | |  |  |
|  | | | | | | | |
| **نعم لا** | | | | | **16-يلتفت للاصوات المختلفه (الناس ، التلفاز أو صوت الشخشيخه)** | **6 شهور** | |
| **نعم لا** | | | | | **17-بيتقلب من علي ظهره الي جانبه** |  |  |
| **نعم لا** | | | | | **18-هل الطفل يلعب بالورق عند إعطائه له مثل ( وضعها بفمه أو يطويها بيده لتصدر أصوات)** |  |  |
|  | | | | | | | |
| **نعم لا** | | | | | **19-يقلق من الغرباء (يبكي ،يبعد ،يحدق)** | **7 شهور** | |
| **نعم لا** | | | | | **20-يهم ليجلس بمفرده من وضع الأستلقاء عندما يمد الأهل يده لهم ؛دون مساعده لشد الطفل.** |  |  |
|  | | | | | | | |
| **نعم لا** | | | | | **21-يضرب الاشياء ببعض أو بالأرض لأصدار أصوات مثل (المكعبات، الاطباق)** | **8 شهور** | |
| **نعم لا** | | | | | **22-يجلس مستقيما دون الحاجه لدعم لعده دقائق.** |  |  |
|  | | | | | | | |
| **نعم لا** | | | | | **23-عند إعطاء الطفل لعبه في يده وهو ممسك بلعبه بيده الأخري هل يظل ممسك باللعبتين .** | **9 شهور** | |
| **نعم لا** | | | | | **24-عندما تمد الأم يديها للطفل وهو جالس هل يحاول شد نفسه ليقف دون مساعده .** |  |  |
| **نعم لا** | | | | | **25-يضحك ،يصدر أصوات عند النظر للمرآه** |  |  |
| **نعم لا** | | | | | **26-يجلس دون مساعده وقادر علي الالتفات ،او يهم ليزحف محاول الوصل للعبه بعيده دون فقد توازنه.** |  |  |
|  | | | | | | | |
| **نعم لا** | | | | | **27- -يلتقط الأشياء الصغيره (بسله او الطعام)مثل الكماشه(بصباعين).** | **10 شهور** | |
| **نعم لا** | | | | | **28- عندما تطلب منه لعبه يمد يده ليعطيها لك حتي لو لم يتركها.** |  |  |
| **نعم لا** | | | | | **29-.يحبى .** |  |  |
|  | | | | | | | |
| **نعم لا** | | | | | **30-هل الطفل يهز الشخشيخه عن قصد لتصدر أصوات.** | **11 شهر** | |
| **نعم لا** | | | | | **31-يمسك الاشياء بسلاسه بواسطه أطراف اصابعه السبابه والوسطي(مثل حبة أرز)** |  |  |
| **نعم لا** | | | | | **32-يجلس دون مساعده** |  |  |
| **نعم لا** | | | | | **33-يقف ممسكا بالاثاث** |  |  |
|  | | | | | | | |
| **نعم لا** | | | | | **34-يعرف بعض الأشياء يأسماءها عندما يسأل عن مكان شئ دون الأشاره اليه ،هل الطفل ينظر إليها.** | **12 شهر** | |
| **نعم لا** | | | | | **35-يقول كلمه من مقطعين با -با ، ما - ما ،نا-نا** |  |  |
|  | | | | | | | |
| **نعم لا** | | | | | **36-يفهم كلمه لا (لا تفعل)، (حطها مكانه، تعالي هنا)** | **13 الى 15 شهر** | |
| **نعم لا** | | | | | **37-يصفق أو يمسك لعبه علي جانبه الايسر بيده اليمني ، يلمس ذراعه الايسر بيديه اليمني، يضع رجل علي رجل وهو نائم علي ظهره.** |  |  |
| **نعم لا** | | | | | **38-يمشي بضع خطوات وهو ممسك بيدين الأم.(لا يعتد بالمشايه او الاثاث الصغير)** |  |  |
| **نعم لا** | | | | | **39-يقلب الصفحات لمشاهدة صور أكثر(أم يحاول قطع الورق فقط)** |  |  |
|  | | | | | | | |
| **نعم لا** | | | | | **40-يردد خلفك بعض الكلمات أو يصدر أصوات شبيها لها** | **16 الى 18 شهر** | |
| **نعم لا** | | | | | **41-يقف دون مساعده لفتره قصيره بعد مساعدته علي الوقوف وأزاله الدعم عنه.** |  |  |
| **نعم لا** | | | | | **42-يشخبط بالألوان** |  |  |
| **نعم لا** | | | | | **43-يرمي الكره بعيداعندما يمسكها بيديه الاثنين.** |  |  |
| **نعم لا** | | | | | **44-هل الطفل يستطيع الوقوف من وضع الأستلقاء الي وضع الوقوف دون أي مساعده خارجيه** |  |  |
| **نعم لا** | | | | | **45-يمشي بضع خطوات بمفرده دون ان يمسك بشئ، حتي ولو كان لا يستطيع الوقوف بمفرده(عندما تساعده علي الوقوف وتركه يستطيع ان يمشي)** |  |  |
| **نعم لا** | | | | | **46-يهز رأسه بالرفض ،يشير لكوب الماء ليشرب ، يشير للعبه يريدها ويبكي لاحضارها** |  |  |
|  | | | | |  |  | |
| **نعم لا** | | | | | **47-يتعرف علي أشياءه مثل(حذائه، لعبته،ملابسه)** | **19 الى 24 شهر** | |
| **نعم لا** | | | | | **48-ينطق كلمتين واضحتين ويعرف معناهم مثل ( بوه،مم)** |  |  |
| **نعم لا** | | | | | **49-يمسك بيد احد أو السور اثناء صعود وهبوط السلم( لا يعتد بالصعود زحفا او جالسا)** |  |  |
| **نعم لا** | | | | | **50-يشير للشئ مع قول اسمه صحيح (كره ) حتي بلغه الطفل** |  |  |
|  | | | | |  |  | |
| **نعم لا** | | | | | **51-يتحدث بجمله مكونه من كلمتين مثل (اريد اشرب ،اريد اكل، بابا خارج )** | **25 الى 30 شهر** | |
| **نعم لا** | | | | | **52- يسمي ثلاثه أشياء بأسمائها مثل صوره كلب، قطه ، حصان، بابا ،ماما)** |  |  |
| **نعم لا** | | | | | **53-يقف علي قدم واحده مثال عند( لبس البنطلون او الحذاء)** |  |  |
| **نعم لا** | | | | | **54-يستطيعا ان يصعد ويهبط دون ان يمسك بالسور** |  |  |

.
