# Supplementary material for: Development and validation of Egyptian developmental screening chart for children from birth up to 30 months
Source: PeerJ. 2020 Nov 11;8:e10301. doi: 10.7717/peerj.10301 (PMC7666562; doi:10.7717/peerj.10301)
Supplement: Supplemental Information 3 [file peerj-08-10301-s003.docx]

|  | **Residence** | | **Total** |
| --- | --- | --- | --- |
|  | **Rural** | **Urban** |  |
| **1.0** | 6 (12.0%) | 44 (88.0%) | 50 (100.0%) |
| **2.0** | 8 (16.0%) | 42 (84.0%) | 50 (100.0%) |
| **3.0** | 11 (22.0%) | 39 (78.0%) | 50 (100.0%) |
| **4.0** | 14 (28.0%) | 36 (72.0%) | 50 (100.0%) |
| **5.0** | 6 (12.0%) | 44 (88.0%) | 50 (100.0%) |
| **6.0** | 9 (18.0%) | 41 (82.0%) | 50 (100.0%) |
| **7.0** | 12 (24.0%) | 38 (76.0%) | 50 (100.0%) |
| **8.0** | 16 (32.0%) | 34 (68.0%) | 50 (100.0%) |
| **9.0** | 13 (26.0%) | 37 (74.0%) | 50 (100.0%) |
| **10.0** | 12 (24.0%) | 38 (76.0%) | 50 (100.0%) |
| **11.0** | 15 (30.0%) | 35 (70.0%) | 50 (100.0%) |
| **12.0** | 12 (24.0%) | 38 (76.0%) | 50 (100.0%) |
| **13.0** | 10 (20.0%) | 40 (80.0%) | 50 (100.0%) |
| **14.0** | 6 (13.6%) | 38 (86.4%) | 44 (100.0%) |
| **15.0** | 13 (22.4% ) | 45 (77.6%) | 58 (100.0%) |
| **16.0** | 2 (4.2%) | 46 (95.8%) | 48 (100.0%) |
| **17.0** | 6 (10.3%) | 52 (89.7%) | 58 (100.0%) |
| **18.0** | 11 (23.4%) | 36 (76.6%) | 47 (100.0%) |
| **19.0** | 2 (4.3%) | 45 (95.7%) | 47 (100.0%) |
| **20.0** | 59 (10.4%) | 43 (89.6%) | 48 (100.0%) |
| **21.0** | 1 (1.9%) | 52 (98.1%) | 53 (100.0%) |
| **22.0** | 4 (8.2%) | 45 (91.8%) | 49 (100.0%) |
| **23.0** | 5 (9.8%) | 46 (90.2%) | 51 (100.0%) |
| **24.0** | 5 (9.6%) | 47 (90.4%) | 52 (100.0%) |
| **25.0** | 2 (4.3%) | 44 (95.7%) | 46 (100.0%) |
| **26.0** | 2 (4.0%) | 48 (96.0%) | 50 (100.0%) |
| **27.0** | 4 (7.7%) | 48 (92.3%) | 52 (100.0%) |
| **28.0** | 2 (4.0%) | 48 (96.0%) | 50 (100.0%) |
| **29.0** | 0 (0.0%) | 50 (100.0%) | 50 (100.0%) |
| **30.0** | 16 (32.0%) | 34 (68.0%) | 50 (100.0%) |
| **Total** | 230 (15.3%) | 1272 (84.7%) | 1503 (100.0%) |

**table 1 : demografic distribution of the sample.**

|  | **Sex** | | **Total** |
| --- | --- | --- | --- |
|  | **Male** | **Female** |  |
| **1.0** | 29 (58.0%) | 21 (42.0%) | 50 (100.00%) |
| **2.0** | 29 (58.0%) | 21 (42.0%) | 50 (100.00%) |
| **3.0** | 25 (50.0%) | 25 (50.0%) | 50 (100.00%) |
| **4.0** | 22 (44.0%) | 28 (56.0%) | 50 (100.00%) |
| **5.0** | 22 (44.0%) | 28 (56.0%) | 50 (100.00%) |
| **6.0** | 25 (50.0%) | 25 (50.0%) | 50 (100.00%) |
| **7.0** | 25 (50.0%) | 25 (50.0%) | 50 (100.00%) |
| **8.0** | 27 (54.0%) | 23 (46.0%) | 50 (100.00%) |
| **9.0** | 25 (50.0%) | 25 (50.0%) | 50 (100.00%) |
| **10.0** | 32 (64.0%) | 18 (36.0%) | 50 (100.00%) |
| **11.0** | 26 (52.0%) | 24 (48.0%) | 50 (100.00%) |
| **12.0** | 28 (56.0%) | 22 (44.0%) | 50 (100.00%) |
| **13.0** | 24 (48.0%) | 26 (52.0%) | 50 (100.00%) |
| **14.0** | 23 (52.3%) | 21 (47.7%) | 44 (100.0%) |
| **15.0** | 32 (55.2%) | 26 (44.8%) | 58 (100.0%) |
| **16.0** | 24 (50.0%) | 24 (50.0%) | 48 (100.0%) |
| **17.0** | 29 (50.0%) | 29 (50.0%) | 58 (100.0%) |
| **18.0** | 28 (59.6%) | 19 (40.4%) | 47 (100.0%) |
| **19.0** | 27 (57.4%) | 20 (42.6%) | 47 (100.0%) |
| **20.0** | 24 (50.0%) | 24 (50.0%) | 48 (100.0%) |
| **21.0** | 31 (58.5%) | 22 (41.5%) | 53 (100.0%) |
| **22.0** | 29 (59.2%) | 20 (40.8%) | 49 (100.0%) |
| **23.0** | 20 (39.2%) | 31 (60.8%) | 51 (100.0%) |
| **24.0** | 28 (53.8%) | 24 (46.2%) | 52 (100.0%) |
| **25.0** | 25 (54.3%) | 21 (45.7%) | 46 (100.0%) |
| **26.0** | 26 (52.0%) | 24 (48.0%) | 50 (100.0%) |
| **27.0** | 27 (51.9%) | 25 (48.1%) | 52 (100.0%) |
| **28.0** | 22 (44.0%) | 28 (56.0%) | 50 (100.00%) |
| **29.0** | 28 (56.0%) | 22 (44.0%) | 50 (100.00%) |
| **30.0** | 23 (46.0%) | 27 (54.0%) | 50 (100.00%) |
| **Total** | 785 (52.2%) | 718 (47.8%) | 1503 (100.0%) |

**table 2 :sex of the sample.**

|  | **Grade on Barada** | | | **Total** |
| --- | --- | --- | --- | --- |
|  | **Normal** | **Less than normal** | **More than normal** |  |
| **1.0** | **48(96.0%)** | **0(0.0%)** | **2(4.0%)** | **50 (100.00%)** |
| **2.0** | **28(56.0%)** | **0(0.0%)** | **22(44.0%)** | **50(100.0%)** |
| **3.0** | **44(88.0%)** | **0(0.0%)** | **6(12.0%)** | **50(100.0%)** |
| **4.0** | **29(58.0%)** | **0(0.0%)** | **21(42.0%)** | **50(100.0%)** |
| **5.0** | **40(80.0%)** | **0(0.0%)** | **10(20.0%)** | **50(100.0%)** |
| **6.0** | **22(44.0%)** | **0(0.0%)** | **28(56.0%)** | **50(100.0%)** |
| **7.0** | **34(68.0%)** | **0(0.0%)** | **16(32.0%)** | **50(100.0%)** |
| **8.0** | **40(80.0%)** | **1(2.0%)** | **9(18.0%)** | **50(100.0%)** |
| **9.0** | **39(78.0%)** | **0(0.0%)** | **11(22.0%)** | **50(100.0%)** |
| **10.0** | **41(82.0%)** | **0(0.0%)** | **9(18.0%)** | **50(100.0%)** |
| **11.0** | **38(76.0%)** | **0(0.0%)** | **12(24.0%)** | **50(100.0%)** |
| **12.0** | **38(76.0%)** | **0(0.0%)** | **12(24.0%)** | **50(100.0%)** |
| **13.0** | **42(84.0%)** | **1(2.0%)** | **7(14.0%)** | **50(100.0%)** |
| **14.0** | **40(90.9%)** | **0(0.0%)** | **4(9.1%)** | **44(100.0%)** |
| **15.0** | **49(84.5%)** | **2(3.4%)** | **7(12.1%)** | **58(100.0%)** |
| **16.0** | **41(85.4%)** | **1(2.1%)** | **6(12.5%)** | **48(100.0%)** |
| **17.0** | **51(87.9%)** | **1(1.7%)** | **6(10.3%)** | **58(100.0%)** |
| **18.0** | **40(85.1%)** | **2(4.3%)** | **5(10.6%)** | **47(100.0%)** |
| **19.0** | **44(93.6%)** | **0(0.0%)** | **3(6.4%)** | **47(100.0%)** |
| **20.0** | **42(87.5%)** | **2(4.2%)** | **4(8.3%)** | **48(100.0%)** |
| **21.0** | **48(90.6%)** | **1(1.9%)** | **4(7.5%)** | **53(100.0%)** |
| **22.0** | **47(95.9%)** | **1(2.0%)** | **1(2.0%)** | **49(100.0%)** |
| **23.0** | **47(92.2%)** | **4(7.8%)** | **0(0.0%)** | **51(100.0%)** |
| **24.0** | **51(98.1%)** | **1(1.9%)** | **0(0.0%)** | **52(100.0%)** |
| **25.0** | **45(97.8%)** | **1(2.2%)** | **0(0.0%)** | **46(100.0%)** |
| **26.0** | **50(100.0%)** | **0(0.0%)** | **0(0.0%)** | **50(100.0%)** |
| **27.0** | **52(100.0%)** | **0(0.0%)** | **0(0.0%)** | **52(100.0%)** |
| **28.0** | **50(100.0%)** | **0(0.0%)** | **0(0.0%)** | **50(100.0%)** |
| **29.0** | **49(98.0%)** | **1(2.0%)** | **0(0.0%)** | **50(100.0%)** |
| **30.0** | **49(98.0%)** | **1(2.0%)** | **0(0.0%)** | **50(100.0%)** |
| **total** | **1278(85.0%)** | **20(1.3%)** | **205(13.6%)** | **1503(100.0%)** |

**table 3: grades of developmental score of egyptian infants on Baroda charts.**

|  | **Socioeconomic** | | **Total** |
| --- | --- | --- | --- |
|  | **moderate** | **high** |  |
| **1.0** | **14 (28.0%)** | **36 (72.0%)** | **50 (100.00%)** |
| **2.0** | **13 (26.0%)** | **37 (74.0%)** | **50 (100.0%)** |
| **3.0** | **11 (22.0%)** | **39 (78.0%)** | **50 (100.0%)** |
| **4.0** | **11 (22.0%)** | **39 (78.0%)** | **50 (100.0%)** |
| **5.0** | **6 (12.0%)** | **44 (88.0%)** | **50 (100.0%)** |
| **6.0** | **12 (24.0%)** | **38 (76.0%)** | **50 (100.0%)** |
| **7.0** | **14 (28.0%)** | **36 (72.0%)** | **50 (100.0%)** |
| **8.0** | **9 (18.0%)** | **41 (82.0%)** | **50 (100.0%)** |
| **9.0** | **9 (18.0%)** | **41 (82.0%)** | **50 (100.0%)** |
| **10.0** | **8 (16.0%)** | **42 (84.0%)** | **50 (100.0%)** |
| **11.0** | **11 (22.0%)** | **39 (78.0%)** | **50 (100.0%)** |
| **12.0** | **10 (20.0%)** | **40 (80.0%)** | **50 (100.0%)** |
| **13.0** | **16 (32.0%)** | **34 (68.0%)** | **50 (100.0%)** |
| **14.0** | **15 (34.1%)** | **29 (65.9%)** | **44 (100.0%)** |
| **15.0** | **15 (25.9%)** | **43 (74.1%)** | **58 (100.0%)** |
| **16.0** | **14 (29.2%)** | **34 (70.8%)** | **48 (100.0%)** |
| **17.0** | **19 (32.8%)** | **39 (67.2%)** | **58 (100.0%)** |
| **18.0** | **11 (23.4%)** | **36 (76.6%)** | **47 (100.0%)** |
| **19.0** | **18 (38.3%)** | **29 (61.7%)** | **47 (100.0%)** |
| **20.0** | **17 (35.4%)** | **31 (64.6%)** | **48 (100.0%)** |
| **21.0** | **19 (35.8%)** | **34 (64.2%)** | **53 (100.0%)** |
| **22.0** | **17 (34.7%)** | **32 (65.3%)** | **49 (100.0%)** |
| **23.0** | **20 (39.2%)** | **31 (60.8%)** | **51 (100.0%)** |
| **24.0** | **20 (38.5%)** | **32 (61.5%)** | **52 (100.0%)** |
| **25.0** | **16 (34.8%)** | **30 (65.2%0** | **46 (100.0%)** |
| **26.0** | **21 (42.0%)** | **29 (58.0%)** | **50 (100.0%)** |
| **27.0** | **16 (30.8%)** | **36 (69.2%)** | **52 (100.0%)** |
| **28.0** | **15 (30.0%)** | **35 (70.0%)** | **50 (100.0%)** |
| **29.0** | **15 (30.0%)** | **35 (70.0%)** | **50 (100.0%)** |
| **30.0** | **15 (30.0%)** | **35 (70.0%)** | **50 (100.0%)** |
| **total** | **427 (28.4%)** | **1076 (71.6%)** | **1503 (100.0%)** |

**table 4: socioeconomic level of the sample.**
